# Co-expression of mFat-1 and pig IGF-1 genes by recombinant plasmids in modified chitosan nanoparticles and its synergistic effect on mouse immunity

**DOI:** 10.1038/s41598-017-17341-x

**Published:** 2017-12-07

**Authors:** Qi Xiong, Jianlin Chen, Fei-Lin Li, Shiji Zhao, Xiaoping Wan, Xiao Yang, Jianglin Li, Danyu Luo, Zezhou Wang, Xuebin Lv, Rong Gao

**Affiliations:** 1 Key Laboratory of Bio-resource and Eco-Environment of Education Ministry, Key Laboratory of Animal Disease Prevention and Food Safety of Sichuan Province, College of Life Sciences, Sichuan University, Chengdu, 610064 Sichuan China; 2 School of Laboratory Medicine, Chengdu Medical College, Chengdu, 610500 Sichuan China; 3 Sichuan Academy of Animal Science, Chengdu, 610066 Sichuan China; 4 Center for Animal Disease Control of Sichuan Province, Chengdu, 610035 China; 5 Chengdu Foreign Language School, Chengdu, 610060 Sichuan China

## Abstract

To develop a cost-effective molecular regulator to improve growth metabolism and immunity of animals, a recombinant plasmid co-expressing fatty acid desaturase (mFat-1) and pig insulin growth like factor 1 (IGF-1) genes was constructed by the 2 A self-cleavage technique. After entrapment within modified chitosan nanoparticles (chitosan modified with polyethyleneglycol–polyethylenimine, CPP), the recombinant plasmid was injected intramuscularly into mice. Compared with controls, co-expression of mFat-1 and IGF-1 significantly raised the level of serum IGF-1, and increased the liver and muscle docosa hexaenoic acid (DHA) content. Th and Tc cell levels were also elevated, as were expression levels of serum IL-4 and IL-6 genes. These results demonstrate that the immunity and metabolism of an animal can be effectively improved by co-expression of mFat-1 and IGF-1 genes *in vivo*, which may contribute to further development of novel immunomodulators with beneficial effects on growth metabolism and immunity.

## Introduction

The *fat-1* gene of *Caenorhabditis elegans* encodes an n-3polyunsaturated fatty acid desaturase that adds a double bond to unsaturated fatty acid hydrocarbon chains. It also convertsn-6 to n-3 polyunsaturated fatty acids (PUFAs), which are absent in most mammals^1,2^. Well recognized for their health benefits, not only cann-3 PUFAs serve as an energy source and a substrate of biochemical reactions, it also exerts effects on inflammatory and autoimmune diseases^3^.

The insulin-like growth factor-1 (IGF-1) is a 70 amino acid peptide^4^ which acts as an anabolic and mitogenic effector of growth hormone, regulating fetal development, growth, and metabolism^5^. IGF-1 in the serum is synthesized mainly in the liver^6^, and exerts its action through its receptor, IGF-1 receptor (IGF-1R). Nearly all immune cells, such as T lymphocytes and B lymphocytes, mononuclear cells and NK-cells, express IGF-1R and are susceptible to IGF-1^7–9^. Alterations inIGF-1 levels in the course of immune and inflammatory reactions can compromise immunity.

While many researchers have focused on the function of *fat-1* and IGF-1 on the growth and metabolism of animals, recombinant *fat-1* and IGF-1 have received less attention. Since the bioactivity of a single recombinant gene may not be sufficient to achieve adequate effects, as a first step in the development of an effective immunoregulator of pigs, we investigated the synergistic activity between these two genes when simultaneously expressed in mouse cells *in vivo*.

## Materials and Methods

### Recombinant plasmid construction

Plasmid VR1012 (Vical company, San Diego, CA, USA) was used as a eukaryotic expression vector. The cDNA of fused mFat-1 and pig IGF-1 genes was constructed from VRmFat-1 and VRIGF-1, recombinant plasmids containing mFat-1 and pig IGF-1 genes respectively.

The 57-base pair 2 A sequence 5′-GGA GAG GGC AGG GGA AGT CTT CTA ACA TGC GGG GAC GTG GAG GAA AAT CCC GGG CCA-3′ encoding amino acids GEGRGSLLTCGDVEENPGP, along with downstream tissue plasminogen activator (TPA) signal sequence, was used to generate a multicistronic cassette linking the fused mFat-1 and pig IGF-1 genes to make a single fragment encoding the proteins^10,11^. The linked fragment was inserted into plasmid VR1012 under control of the human cytomegalovirus(CMV)promoter using standard methods. The recombinant plasmid was validated by sequencing and named VRMAG, the recombinant VR1012 plasmid containing fused mFat-1, 2 A and pig IGF-1 genes.

### Large-scale preparation of plasmid DNA

*Escherichia coli* (*E*. *coli*) DH5α cells were transfected with the recombinant plasmid and plated on Luria Bertani (LB) medium with 60 μg/ml kanamycin (Kana). Positive clones were screened after incubating in 37 °C and confirmed by PCR assay.

Plasmid VRmFat-1, VRIGF-1, VRMAG and VR1012 were used to inoculate 400 ml LB broth with Kana at 60 µg/ml and shaken for 16 h at 37 °C. Bacterial cells were pelleted by centrifugation and plasmid DNA extracted following large-scale alkaline lysis. The plasmid was precipitated and purified by the spermine method^12^. Residual contamination by endogenous toxin of *E*. *coli* was < 0.1 EU/mg plasmid as measured by the Limulus amebocyte lysate test^13^. Plasmid DNA was resuspended in sterile water and stored at −20 °C until use^14^.

### Preparation and detection of recombinant plasmids encapsulated in CPP nanoparticles

Chitosan methoxypoly(ethylene glycol)-polyethylenimine(CPP) was prepared from chitosan as described by Xu.^15^ and nanoparticles of CPP complexes were prepared by the ionotropic gelation method as described elsewhere^16^. Briefly, 30 mg CPP dissolved in CH3COOH/CH3COONa (pH 5.5), and 1 mg plasmid DNA solution (with 10 µl 10 mg/ml Na tripolyphosphate solution) were heated in a 55 °C water bath for 5 min. An equal quantity of each plasmid solution was then gently mixed with CPP (plasmid and CPP mass ratio 1:30) and left for 5 min to form VRMAG-CPP, VRmFat-1-CPP, VRIGF-1-CPP and VR1012-CPP. The resulting CPP particles were observed by transmission electronic microscopy, and the granule diameter, dispersity and zeta potential were analyzed by Zeta sizer 3000 HS/IHPL (Malvern Instruments Ltd., Malvern, UK)^17^.

### Animal procedures

Forty healthy 21-day old (20–30 g) female Kunming mice were purchased from Dashuo Biotechnology Company, Chengdu, and were randomly divided into four groups (C, A1, A2, A3), 10 per group. Each was injected intramuscularly with 0.1 mg encapsulated plasmid: the mice in group C(control) with VR1012-CPP, group A1 withVRIGF-1-CPP, A2 withVRmFat-1-CPP, and A3 with VRMAG-CPP. EDTA-K_2_ stabilized blood samples (~20 µL) were collected from the tail vein and pooled within groups for determination of immune cells, immunoglobulin titers and expression levels of interleukin genes at 0, 1, 2, 3 and 4 weeks post-injection. Animal experiments were performed according to Chinese animal welfare laws and regulations, and approved by the Institutional Animal Care and Use Committee of Sichuan University, Sichuan, China under permit No. SCUBC20140305.

### Assay of IGF-1—sandwich ELISA

IGF-1 ELISA kits (eBioscience, San Diego, CA, US) were used to assay serum IGF-1 according to the manufacturer’s protocol. Briefly, serum samples of were added to microtitration plates pre-coated with monoclonal antibody to mouseIGF-1. Standard curves were constructed from serial 2-fold dilutions, in triplicate, of recombinant mouse IGF-1. ODs were measured at 450 nm in a microplate reader 680 (Bio-Rad, California, USA), Wells without mouse serum were included as a negative control.

### Assay of DHA—GC/MS

At 5-weeks post-injection, the mice were euthanized by cervical dislocation and liver, muscle and intestinal adipose tissues were excised. Group samples of each tissue were pooled separately and stored at −80° until used. Methods for extraction and separation of lipids, and for the preparation of fatty acid methyl esters, have been described^17^. Analysis of fatty acid methyl esters by gas chromatography was carried out using a capillary column (CP-Sil88 FAME 100 m × 0.25 mm × 0.20 um, Supelco, Varian, USA) with flame ionization detection. The injector and detector were maintained at 250 °C and 260 °C, respectively. The initial column temperature of 110 °C was held for 4 min then raised at 3 °C/min to 170 °C followed by a ramp of 2 °C/min to 200 °C. The final temperature was held for 6 min. Total ionmonitoring was performed, encompassing mass ranges from 50–550 atomic mass units. DHA (22:6 n-3) mass was determined by comparing areas of DHA to that of a fixed concentration of internal standard.

### Immunological assays

#### Measurement of Th and Tc cells by flow cytometry

Mouse anti-mouse CD4 and CD8a (Lγ-2) mAbs, labeled with fluorescein isothiocyanate (FITC) and phycoerythrin (PE) respectively, were purchased from eBioscience (USA). 20 μl venous bloods were mixed with 30 μl normal saline, and incubated with 0.4 μl FITC-conjugated anti-CD4, 0.4 μl PE labeled anti-CD8a and 2.2 µl PBS for 20 min in the dark. 2 ml (10% v/v) lysing solution (Becton Dickinson, USA) was then added for 5–10 min to ensure complete lysis of erythrocytes, and surviving cells were washed twice with PBS, with centrifugation between each step for 5 min at 500 g. Finally, the cells were resuspended in 100 μl PBS and analyzed in a FACScan flow cytometer (Becton Dickinson).

#### Assays of IgG, IgG1 and IgG2a—sandwich ELISA

Mouse IgG, IgG1 and IgG2a quantitation ELISA kits were purchased from eBioscience (USA). Briefly, 96-well flat-bottomed plates were coated with capture antibodies, goat anti-mouse IgG, IgG1 and IgG2a and blocked with blocking buffer. Purified reference mouse serum was diluted in sample diluent at various concentrations to produce a standard curve, which was included for each plate; and serial dilutions of mouse serum samples were added such that at least one value fell within the range of the standard curve. Goat anti-rat secondary antibody was applied after washing. Tetramethyl benzidine (TMB) was added as substrate, and absorbance was measured at 450 nm in a microplate reader 680 (Bio-Rad).

#### Quantitative real-time polymerase chain reaction (QRT-PCR)

Blood samples in each group were pooled (about 100 μl), centrifuged for 5 min at 1,500 g, and mixed with 500 μl RNAiso Plus(TaKaRa Biotechnol., Japan) stored at −80 °C until used. Total RNA was extracted and reverse-transcribed at 37 °C for 60 min as described (TianGen, Quantscript RT kit).

Reverse-transcription PCR was performed using Transcript One-Step gDNA Removal and cDNA synthesis SuperMix (Transgen, Beijing). Real-time PCR was performed on the iQ5 (BIO-RAD) in a total reaction volume of 15 μL, consisting of 7 μL cDNA, 7.5 μL SsoFast EvaGreen SuperMix (BioRad, Singapore), and 0.25 μL each of forward and reverse primers. The standard QRT-PCR protocol was 95 °C for 3 min before the 1st cycle, 95 °C for 10 s, and 58 °C for 20 s, repeated 40 times), and a negative control with no added template was included in each run. Primer sequences, their annealing temperatures and amplification efficiency are listed in Table 1. Beta-actin was used as the reference gene, and the relative expression of immune genes was calculated by the geometric means method and the formula: relative level = 2^−ΔΔCt^ ^18^.

**Table 1:** Primers for Real-Time qPCR.

Gene	Sequence(5′-3′)	Annealing Temperature (°C)
β-actin^1^	F^2^: TACGCCAACACGGTGCTGTC	58.0
	R^3^: GTACTCCTGCTTGCTGATCCACAT	
IL-4	F: GCTGCCCCAGAGAACACGAC	61.0
	R: AGGTTCCTGTCAAGTCCGCTC	
IL-6	F: TGCAATGAGAAAGGAGATGTGTG	56.5
	R: CCCAGATTGGAAGCATCCGT	

^1^The primer of β-actin is designed as reference gene. ^2^Forward primer. ^3^Reverse primer.

### Statistical analysis

Data from all groups were statistically compared by the statistical software program Systat 10 (SPSS). Differences between the groups were analyzed by two-way ANOVA and Turkey multiple comparison. Data were considered to be significantly different when P < 0.05.

## Results

### Properties of CPP nanoparticles

Observation by transmission electron microscopy revealed that most of the CPP nanoparticles were spherical. Analysis by Zetasizer3000 HS/IHPL showed that the average granule diameter was 166 ± 2.53 nm (range 80 to 300 nm), and the polydispersity index of nanoparticles was 0.195; zeta electricity potential was +20.36 mV, indicating that the nanoparticles were positively charged.

### IGF-1 levels

**Figure 1:**
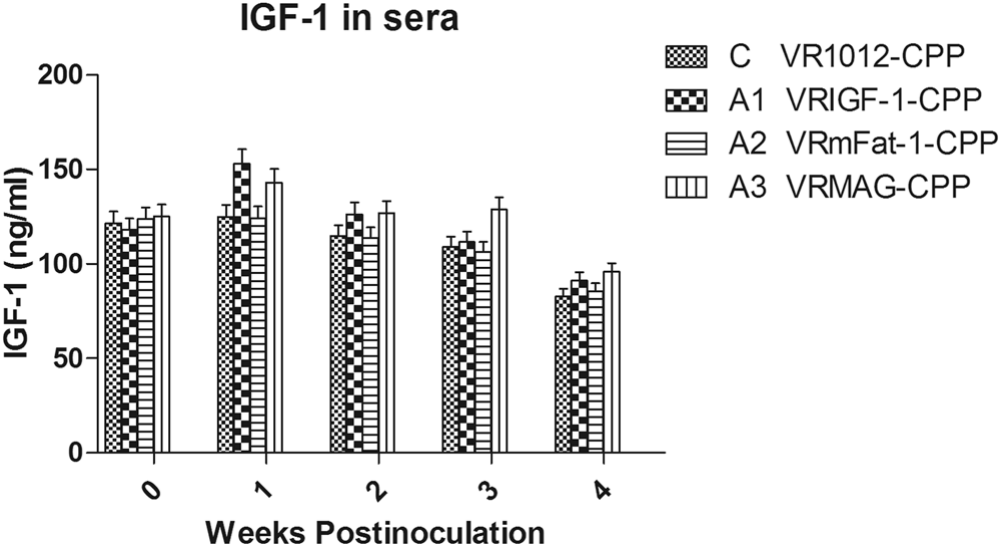
Levels of IGF-1 in the sera of treated mice.

Figure 1 shows the levels of IGF-1 were significantly increased in the sera of groups A1 and A3 compared with groups C and A2 (P < 0.05), and not significantly different in groups A1 and A3 (P > 0.05), and groups C and A1 (P > 0.05).

### DHA levels

**Figure 2:**
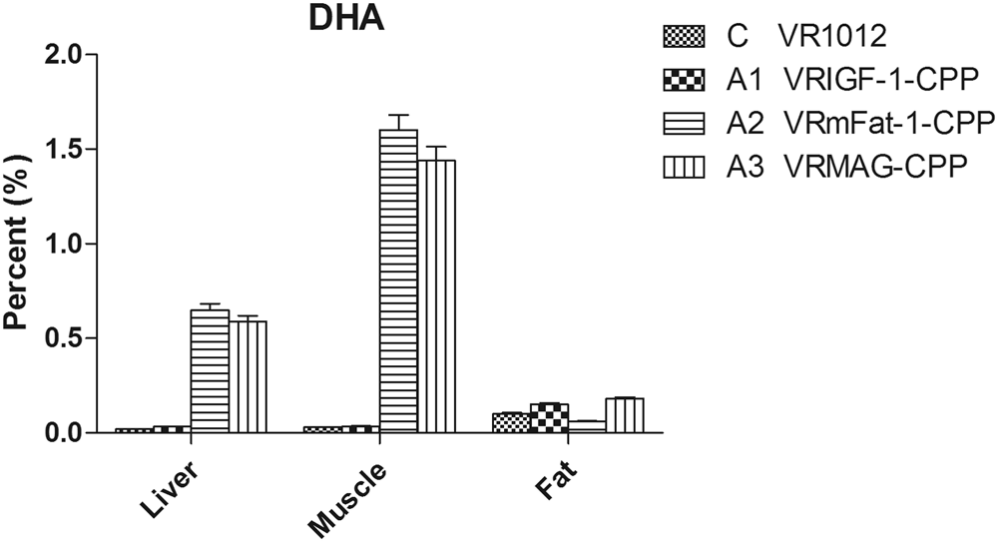
DHA levels in different tissues of treated mice.

To evaluate fatty acid metabolism of inoculated mice, the dynamic changes in DHA contents were determined over the 4 week test period (Fig. 2). DHA levels were significantly increased in liver and muscle tissue of groups A2 and A3 compared with group C and A1 (P < 0.05), but not significantly between group A2 and A3 (P > 0.05), or group C and A1 (P > 0.05).

### Th and Tc levels

**Figure 3:**
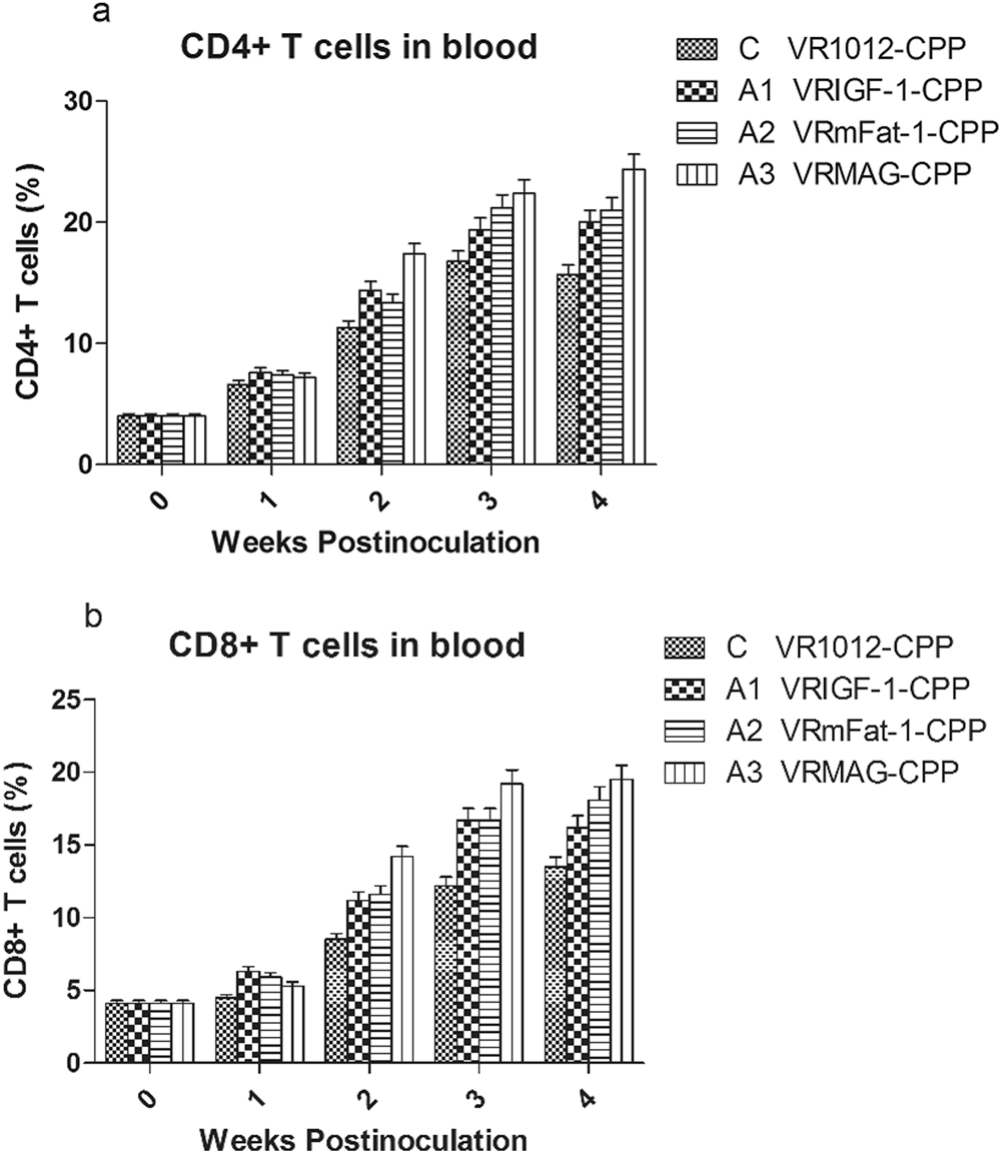
T_H_ and T_C_ cells levels in the blood of treated mice.

Figure 3 shows that levels of CD4+ and CD8+ T cells were significantly increased in the A1, A2 and A3 group compared with C group (P < 0.05) from 2 to 4 weeks after treatment. CD4+ and CD8+ T cells were also increased in the VRMAG group compared with the other groups although not significantly (P > 0.05).

### Change of the level of IgG, IgG1 and IgG2a

**Figure 4:**
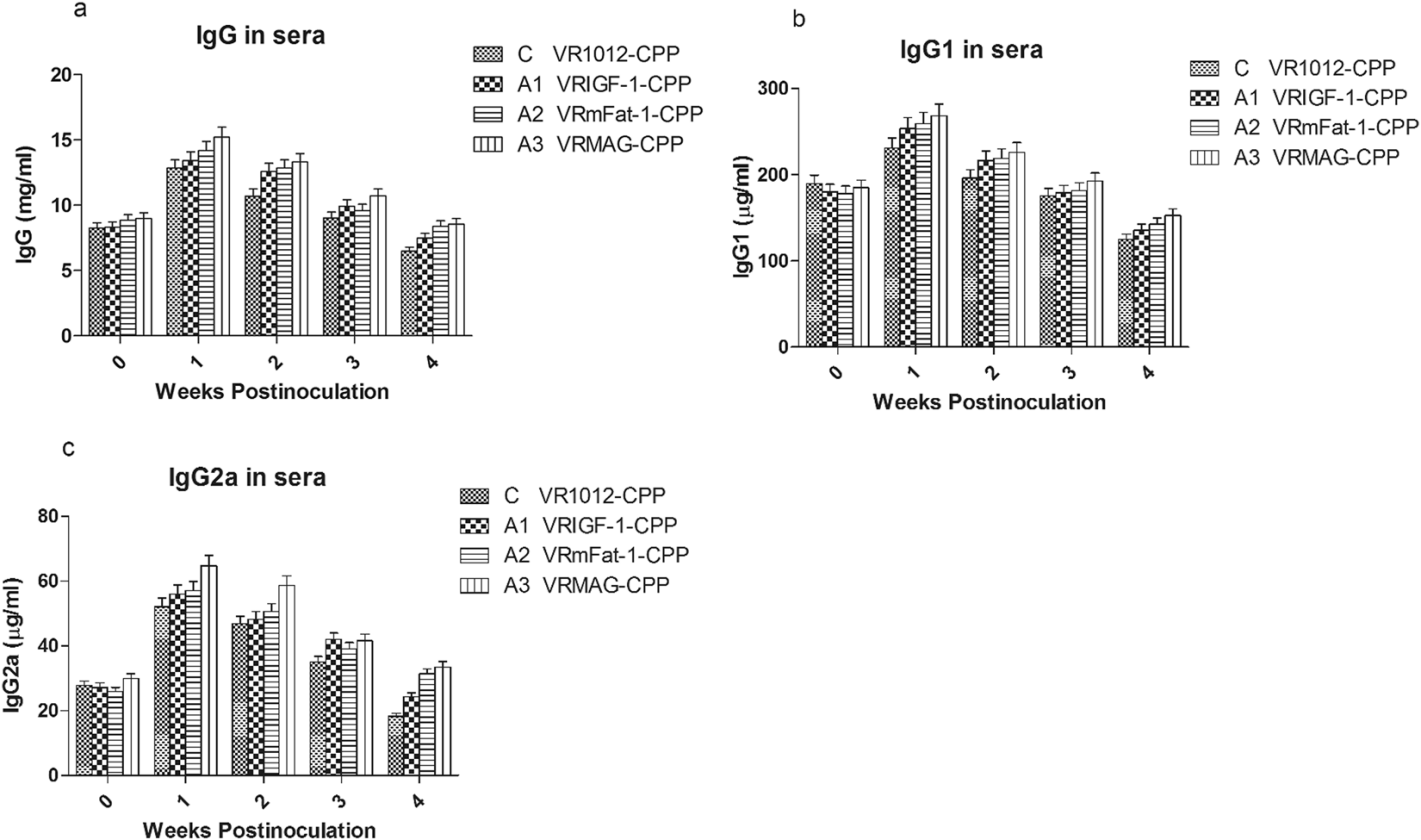
Immunoglobulins content in the sera of treated mice.

Figure 4 shows that levels of IgG, IgG1 and IgG2a were significantly increased in the sera of group A1, A2 and A3 following treatment (P < 0.05), with group A3 significantly higher than group A1 (P < 0.05). Changes in IgG, IgG1, and IgG2a between group A1/A3 and group A2 were not significant (P > 0.05).

### Interleukin gene expression

**Figure 5:**
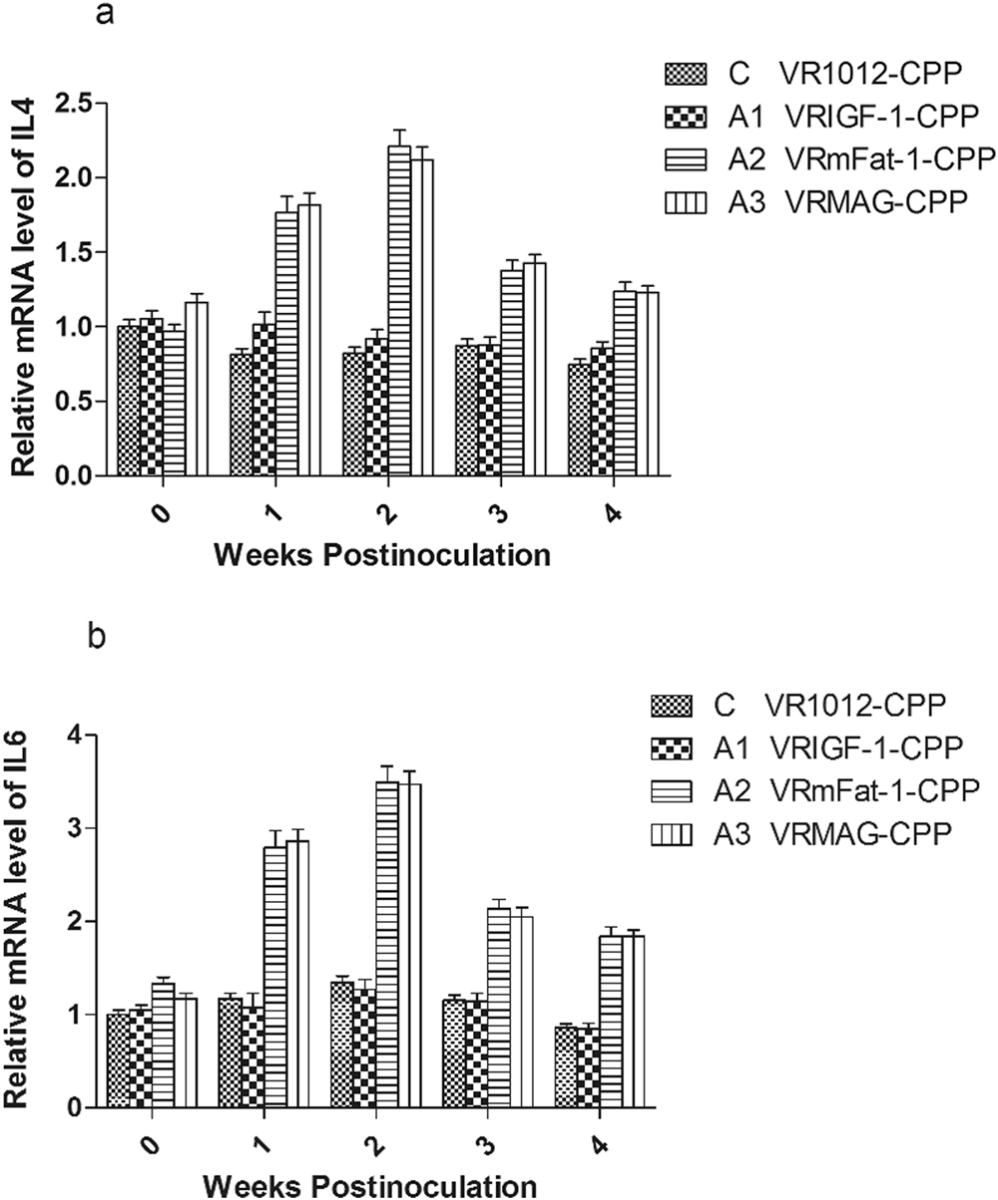
Expression levels of IL-4 and IL-6 in the blood of treated mice.

Using pre-treatment cDNA data as the standard template, the efficiency of PCR amplification of the immune genes ranged from 95–105%. Figure 5 shows that expression of IL-4 and IL-6 genes significantly increased in A2 and A3 groups from 1 to 4 weeks post-treatment compared to controls (P < 0.05), and differences between A2 and A3 were not significant (P > 0.05), and so were the differences between A1 and C (P > 0.05).

### Weight change of mice

Growth rates of treated mice, especially the A3 group, were significantly higher than controls over the 4-weeks test period (Table 2).

**Table 2:** Changes in mouse weights during the 4 weeks of observation (n = 10/group).

Group	Initial weight (g)	End weight (g)	Net gain (g)	Average gain (g)
C	25.3 ± 2.16	29.6 ± 2.14	4.3 ± 2.06	0.86^a^
A1	25.7 ± 3.27	31.8 ± 4.92	6.1 ± 3.21	1.22^b^
A2	25.3 ± 2.87	31.3 ± 3.38	6.0 ± 2.44	1.20^b^
A3	25.3 ± 2.54	32.4 ± 3.14	7.1 ± 2.76	1.42^c^

Note: Different superscripts indicate significant differences (P < 0.05).

## Discussion

Previous studies have demonstrated that the *fat-1* and IGF-1 genes are important for regulation of growth and metabolism. Fat-1catalysesn-6 to n-3PUFAs that are essential dietary fatty acids for mammals as they lack desaturases and elongation enzymes^1–3^. Studies have shown that n-3 PUFA can prevent diabetes and tumors^19,20^, offering potential treatment of insulin resistance and diabetes^21^, and suppression of liver tumorigenesis by down regulating TNF-α^22^. In addition, an increased intake of dietary n-3PUFAs has been shown to improve cardiovascular, neoplastic and neurodegenerative diseases, including Alzheimer’s disease (AD)^23^. IGF-1 is a critical regulator of many physiological functions, ranging from longevity to immunity. It has been shown that IGF-1is the major mediator of GH-stimulated somatic growth and a mediator of GH-independent anabolic responses in many cells and tissues^24,25^. IGF-1 is also involved in the regulation of hematopoiesis^26^, and the diverse functions of cells of the innate and acquired immune systems^17,27^. It exerts an inhibitory action on inflammatory and Th1-mediated cellular immune responses through stimulation of IL-10 production in T cells^28^, and it may also enhance the proliferation of T cells^29^.

In this report, the mFat-1 and IGF-1 genes were co-expressed by use of the self-cleavage 2 A peptide technique, which allows multiple genes to be equally expressed within the same vector *in vivo* ^10,30^ in order to develop a novel and effective molecular regulator to improve growth metabolism and immunity. Importantly, during the whole experimental period, no local lesions or injury (injection inflammation) or systemic symptoms were observed in any of the mice during the 4-week sex perimental period. Our results indicate that levels of IGF-1 in the sera of mice treated with recombinant plasmid VRMAG were significantly increased, as was the content of DHA in liver and muscle tissue. This may the reason for VRMAG group mice had a better growth gain during 4 weeks of observation. These results suggest that VRMAG can produce stronger and beneficial regulation of growth metabolism than the VRIGF-1 and/or VRmFat-1 alone.

Notably, our experiments found that the content of IgG, IgG1 and IgG2a in sera significantly increased in the treated groups, and VRMAG mice showed optimal humoral immunity improvement. Moreover, the amounts of CD4+ T cells and CD8+ T cells were significantly elevated in the VRMAG group in comparison with that of the control group. Similarly, the expression levels of IL-4 and IL-6 genes also significantly increased in the VRMAG and VRmFat-1 treated mice post injection. We noted that there is no significant difference between VRMAG and VRmFat-1 group. This may be explained by that IGF-1 have no significant effect on IL-4 and IL-6 expression levels compared with control, although IL-4 can stimulate IGF-1 production^31^. We have previously shown that pig IL-4 and IL-6 genes can be safely applied to potentiate the systemic immunity of animals against disease^32^.

As our previous study shown that the CPP prepared nanoparticles for gene delivery had little toxicity on the growth and gene expression^15^, and the blank vector plasmid group have no difference with the blank group^14,33^. From Figs 1 to 5 it will be noted that the control values did not remain constant over the 4-weeks test period. This may be explained by normal growth changes occurring in the young mice.

## Conclusions

As a first step for development effectively immunoregulator of pigs, our results demonstrate that VRMAG-CPP can effectively improve growth metabolism and, compared with the two genes separately, can induce a synergistic enhancement of systemic humoral and cellular immunity of mice. Future work will be to determine if VRMAG will enhance immunity against infectious diseases in pigs.
